# Effectiveness of evidence-based decision aids for women with pathogenic *BRCA1* or *BRCA2* variants in the german health care context: results from a randomized controlled trial

**DOI:** 10.1186/s12911-023-02327-9

**Published:** 2023-10-16

**Authors:** Sibylle Kautz-Freimuth, Marcus Redaèlli, Arim Shukri, Hannah Kentenich, Dusan Simic, Vanessa Mildenberger, Rita Schmutzler, Kerstin Rhiem, Stephanie Stock

**Affiliations:** 1 Institute of Health Economics and Clinical Epidemiology, Faculty of Medicine and University Hospital Cologne, University of Cologne, Gleueler Straße 176-178, 50935 Cologne, Germany; 2 Center for Familial Breast and Ovarian Cancer, Center for Integrated Oncology (CIO), Faculty of Medicine and University Hospital Cologne, University of Cologne, Kerpener Straße 62, 50937 Cologne, Germany

**Keywords:** *BRCA1* and *BRCA2* variants, Decision aids, Decisional conflict, Decision-making, Hereditary breast and ovarian cancer, Preventive options, Preference-sensitive decisions

## Abstract

**Background:**

Women with pathogenic *BRCA1* or *BRCA2* variants are at high risk for breast and ovarian cancer. Preventive options include risk-reducing breast and ovarian surgeries and intensified breast surveillance. However, individual decision-making is often associated with decisional conflicts. Two evidence-based decision aids have recently been developed for these women (healthy or with unilateral breast cancer) for the German context to support them in their decision-making process. This study evaluated their effectiveness.

**Methods:**

In a randomized controlled study, women (aged 18–70 years) with pathogenic *BRCA1* or *BRCA2* variants were randomly assigned 1:1 to the intervention (IG, n = 230) or control (CG, n = 220) group. All participants received usual care. After baseline survey (t0), IG participants additionally received the DAs. Follow-up surveys were at three (t1) and six (t2) months. Primary outcome was decisional conflict at t1. Secondary analyses included decision status, decision regret, knowledge on risks and preventive options, self-reported psychological symptoms, acceptability of DAs, and preparation for decision-making.

**Results:**

Of 450 women recruited, 417 completed t0, 398 completed t1 and 386 completed t2. Compared to CG, IG participants had lower decisional conflict scores at t1 (p = 0.049) and t2 (p = 0.006) and higher scores for knowledge (p = 0.004), acceptability (p = 0.000), and preparation for decision-making (p < 0.01).

**Conclusions:**

These DAs can help improve key parameters of decision-making in women with pathogenic *BRCA1* and *BRCA2* variants and, thus, provide a useful add-on to the current counseling and care concept for these women in Germany.

**Trial registration:**

German Clinical Trials Register, DRKS-ID: DRKS00015823, retrospectively registered 14/06/2019.

## Background

Women with pathogenic germline variants in the *BRCA1* or *BRCA2* gene have an increased lifetime risk of breast cancer (BC) and ovarian cancer (OC), compared to the general female population, with cancer occurring about twenty years earlier than the sporadic forms. The average cumulative lifetime risk of women without a history of cancer (previvors) is about 70% for BC and 44% (*BRCA1* variant) or 17% (*BRCA2* variant) for OC [1]. The corresponding risks in the general female population in Germany are about 12.4% for sporadic BC and 1.3% for sporadic OC [2]. Women with a history of unilateral BC (survivors) have an increased risk of contralateral BC, ranging from about 40–44% (*BRCA1* variant) and 26–33.5% (*BRCA2* variant) [1, 3].

In Germany, women with newly identified *BRCA1* or *BRCA2* variants receive individual care and counseling on their genetic findings, cancer risks, and preventive options at the centers of the German Consortium of Familial Breast and Ovarian Cancer (GC-HBOC). To address their BC risks, previvors are offered intensified breast surveillance (IBS) or risk-reducing bilateral mastectomy (RRBM). IBS can detect BC at an early, potentially curable stage in more than 80% of cases [4], but fails to reduce the risk of developing BC. RRBM reduces the risk of developing BC to approximately 2% [5] and may provide a survival benefit for women with pathogenic *BRCA1* variants [6]. Yet, RRBM represents an irreversible decision affecting physical sensation, breastfeeding ability, or emotional well-being, among other things and has not been shown to provide a survival benefit in women with pathogenic *BRCA2* variants [6]. Survivors can opt for IBS including aftercare (IBSA) or risk-reducing contralateral mastectomy (RRCM). RRCM significantly lowers contralateral BC risk and overall mortality [7]. However, this option is limited in the presence of competing risks, such as a high recurrence risk in the affected breast, which must be weighed against the RRCM benefits. Since effective methods for early OC detection are missing, both previvors and survivors are only offered risk-reducing bilateral salpingo-oophorectomy (RRBSO) [8]. RRBSO significantly reduces OC morbidity [9] and OC specific and overall mortality [10], but leads to loss of fertility and may cause surgical menopause with consequences such as osteoporosis, cardiovascular diseases, or menopausal symptoms [11]. Alternatively, women may also choose to wait and see in the first step and not take any of the options initially.

Given these multiple options including pros and cons, women with pathogenic *BRCA1* and *BRCA2* variants face complex considerations and far-reaching, life-changing decisions. They need to gain clarity on how they feel about their individual future cancer risks at their current stage of life and age, weigh which consequences and potential adverse effects they are most likely to accept, and consider what option they want to choose and when. Additionally, they need to make further decisions, e.g. on breast reconstruction after mastectomy or on family planning. Each person evaluates the benefits and risks of each preventive option differently, depending on one’s own values and preferences. Furthermore, in healthy women with pathogenic *BRCA2* variants, neither option with respect to BC risk (IBS or RRBM) has yet shown medical superiority in terms of overall survival. Thus, women with pathogenic *BRCA1* and *BRCA2* variants face preference-sensitive decisions [12]. These may cause decisional conflicts, which may result e.g. in decision delay, dissatisfaction or decision regret [13, 14].

To support persons in their complex decision-making process and help them make high-quality decisions, informed decision-making should be encouraged. This means, they must have sufficient knowledge about the available options and make their decision in accordance with their personal values and preferences [15]. To this end, evidence-based decision aids (DAs), used in addition to medical advice, are valuable tools. DAs have been shown to effectively support decision-making and improve decision quality by increasing knowledge about options for action, promoting a realistic estimation of benefits and risks, increasing agreement between one’s own values and the decision made, and decreasing decisional conflict and unclarity about one’s own values [16].

In a previous research phase, we developed two evidence-based DAs for previvors and survivors in the German healthcare context according to the International Patient Decision Aid Standards (IPDAS) [17]. Following to IPDAS requirements, the present study aims at evaluating the DAs compared to usual care (UC) in terms of decision- and knowledge-related outcomes, self-reported psychological symptoms, user acceptability and usefulness of the DAs for preparing decision-making [18].

## Methods

### Study design

The study was designed as a monocentric RCT and conducted at the Center for Familial Breast and Ovarian Cancer at the University Hospital Cologne, Germany [registered as DRKS00015823]. Enrollment started in January 2019 and ended in October 2021. Data were collected at baseline (t0), three months (t1), and six months (t2) after study inclusion. The study protocol has been published elsewhere [18]. Prior to study initiation, the project was approved by the Ethics Committee of the Faculty of Medicine of the University of Cologne, Germany [ethical approval dated 26 April 2017, reference number 17–128].

### Study population

Women who met the inclusion criteria for genetic testing consented by the GC-HBOC [19], who had received their genetic test result and who indicated during the informative talk about the study that they were not yet fully decided regarding their final irrevocable prevention option(s) were enrolled. The latter criterion included women who indicated (1) having not yet decided at all, (2) having not yet decided on least one final, irrevocable option (RRM or RRBSO), and (3) having made primary decisions but wanted them vetted through evidence-based decision-making. Additional inclusion criteria were informed consent to study participation, adequate knowledge of the German language, and no medical contraindication to potential risk-reducing surgery. Exclusion criterion for previvors was any cancer history. Survivors must not have advanced BC (e.g., local recurrence, distant metastases), and other cancers except unilateral BC.

Eligible women were recruited by specialist physicians following post-test genetic counseling (PTGC) or during a consultation at IBS/IBSA. Two study nurses provided organizational support. After obtaining written informed consent, participants were enrolled in the study and randomly assigned to the intervention group (IG) or control group (CG) in a 1:1-ratio. All participants received a pseudonymized baseline questionnaire t0 and were asked to return the completed questionnaire within two weeks.

### Intervention and control

All participants received UC, offered at the GC-HBOC centers following genetic testing and established as the current gold standard. A detailed description of UC is given elsewhere [18]. Briefly, UC includes counseling regarding gene variant, personal risk profile including further genetic and non-genetic risk factors, individual future risks for BC/OC, inheritance and risks to family members, and preventive options offered in German health care [8, 20].

The intervention was an evidence-based DA for women with pathogenic *BRCA1* or *BRCA2* variants. There was a modification each for previvors and survivors, each with target group-specific information where needed. The development process and the final contents are described elsewhere [17]. After return of the completed baseline questionnaire (t0), the respective DA, used as a printed A5-format booklet, was mailed to IG participants. CG participants did not receive a DA or any intervention other than provided by UC.

### Outcomes

The primary outcome was the extent of decisional conflict (total score) three months after study inclusion (t1). Secondary outcomes at t1 were decision status, knowledge, self-reported symptoms of anxiety, depression, and distress, DA acceptability and usefulness of the DA for preparation of decision-making. Secondary outcomes at t2 were decisional conflict, decision status, decision regret, and self-reported symptoms of anxiety and depression.

### Data collection instruments

Decision-related data were collected using the decisional conflict scale (DCS [21, 22]), the stage of decision-making scale (SDM-S [23, 24]), and the decision regret scale (DRS [14, 25]). The DCS includes five subscales (informed, values clarity, support, uncertainty, effective decision) with a total of 16 items to be rated on a five-point Likert scale (from 1 = strongly agree to 5 = strongly disagree) which are summed to a total score. The DRS consists of five items to be rated on a five-point Likert scale (from 1 = strongly agree to 5 = strongly disagree). The achievable scores for DCS and DRS range from 0 (extremely low) to 100 (extremely high). The SDM-S version used consists of one item with the following four response options: (1) “I have not yet thought about the options”, (2) “I am considering the options”, (3) “I am close to choosing one option”, and (4) “I have already made a choice”. To determine decision status, the proportion of women who assigned themselves to one of the three SDM-S phases for “not yet decided” (= undecided, answers (1) to (3)) was compared with the proportion of those who classified themselves as “decided” (= decided, answer (4)).

Knowledge-related outcomes were assessed with an instrument containing 15 statements about BC/OC risks and prevention options, each to be rated as “agree,“ “disagree,“ or “don’t know.“ Previvors and survivors received target group-specific statements, some of which (n = 6) therefore differed. The number of correct answers was used to determine the knowledge level: For each participant, each correct answer was coded 1 and each incorrect answer was coded 0. Then a knowledge sum score was formed.

Self-reported psychological outcomes were collected with the hospital anxiety and depression scale (HADS [26, 27]) and the impact of event scale-revised (IES-R [28, 29]). Achievable scores for the HADS subscales anxiety and depression, each comprising seven items, range from 0 (extremely low) to 21 (extremely high). Scores below 8 are defined as non-cases [30]. The IES-R consists of 22 items that are rated using a four-point-scale (from 0 = not at all to 5 = often). The achievable scores for the IES-R subscales intrusion, hyperarousal, and avoidance range from 0 (extremely low) to 35 or 40 (extremely high), respectively. The IES-R also can also provide an indication that post-traumatic stress disorder (PTSD) may be present. Using the respective score algorithm, a score above 0 may indicate PTSD [29].

Acceptability and usefulness for decision-making preparation of the DA were assessed with (1) an adapted acceptability instrument from O’Connor & Cranney [31] and used by Metcalfe [32], and (2) the preparation for decision-making scale (PrepDMS [33, 34]). The acceptability instrument consists of six items. Positively rated items were coded 1 and a total score was built per participant. Acceptability total score ranges from 0 (low) to 6 (high). Additionally, IG participants were asked, if they would recommend the DA to other women in a similar situation. The PrepDMS consists of a total of ten items which are rated on a five-point Likert scale (from 1 = not at all to 5 = a great deal). It includes two subscales related to preparation for decision-making and for the physician consultation, and the total score. PrepDMS scores range from 0 (not helpful at all) to 100 (extremely helpful). To rate both acceptability and usefulness for decision-making preparation (PrepDMS), IG participants were asked to rate the DA, CG participants were asked to rate the written material provided as part of UC.

### Statistical analysis

The sample size was calculated based on previous studies [32, 35, 36]. A conservative assumption was made with a small effect size (Cohen’s d) of 0.3, an α of 0.05, and a ß of 0.2 or a power of 0.8 (1-ß), respectively. As numerous studies show that DAs significantly reduce decisional conflict [16, 32, 37], one-sided testing (*t*-test) of decisional conflict (total score) at t1 was conducted. This required an actual sample size of n = 155 participants per group, including a mean dropout rate of about 10% in previous evaluation studies [38, 39]. Little’s test for Missing Completely at Random (MCAR) was performed to determine whether imputation of missing values is required. To assess internal consistency, Cronbach’s alpha was calculated for each scale. Analysis of baseline data (t0) was to verify comparability of both study groups. Outcomes were measured at t1 and t2. Metric data were described by mean, standard deviation, median, minimum, and maximum. Categorical data were described by frequencies and percentages. The number of non-missing values was also reported. Mean differences of metric variables between IG and CG were tested with the independent two-sided (one-sided only with DCS total score at t1) *t*-test in the presence of normal distribution. Mean differences of scores within groups between follow-ups were assessed with the dependent two-sided *t*-test. Nonparametric tests were used in the presence of non-normal distribution. Differences in categorical variables were tested using the chi-square test. Data were analyzed using IBM SPSS Statistics for Windows, version 27.0 (IBM Corp: Armonk, New York) and R [40]. For all statistical tests, a α-level of 0.05 was considered significant.

## Results

**Fig. 1:**
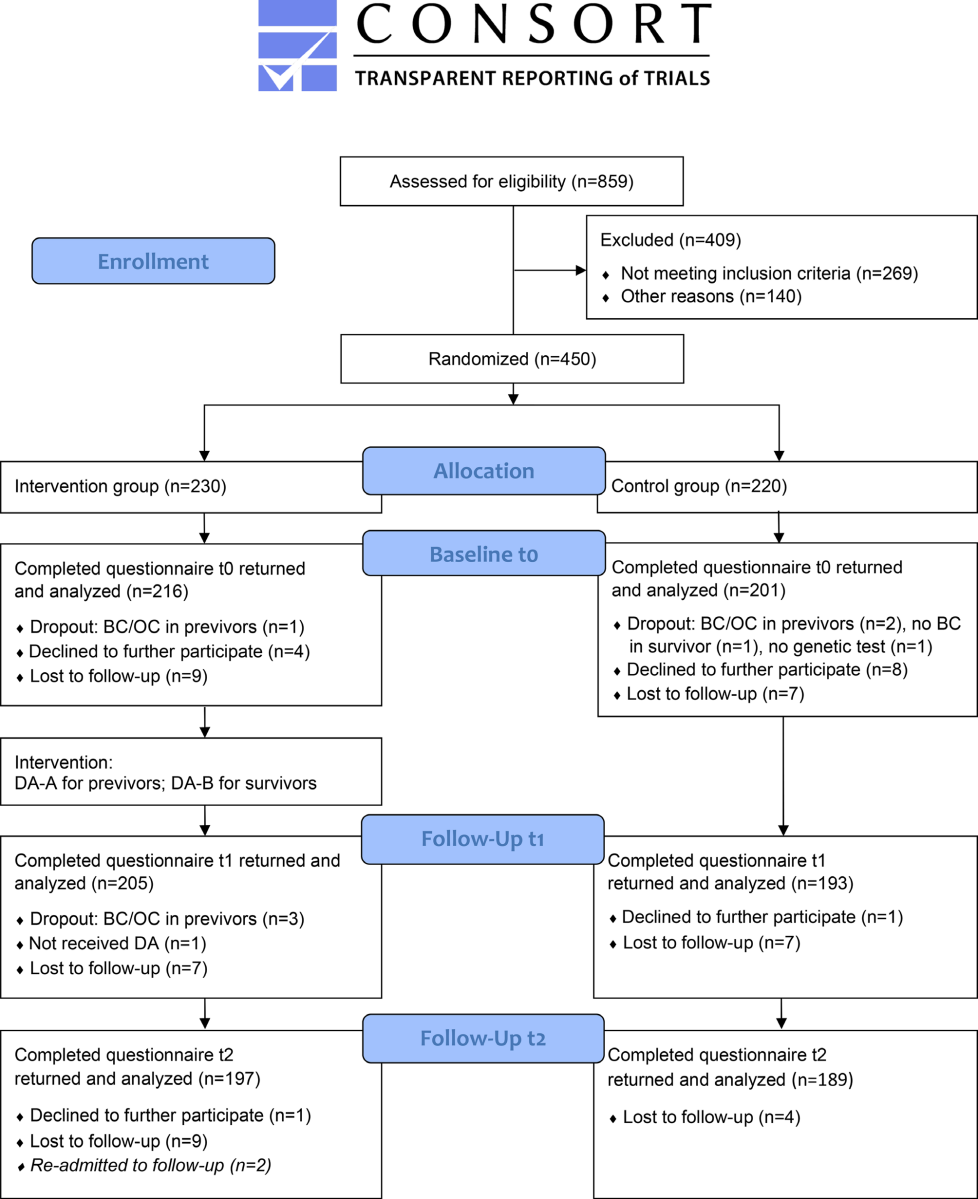
Study flow according to the CONSORT 2010 Flow Diagram

Figure 1 provides an overview of the study flow and shows that the dropout rate was similar in both groups. As Little’s test for MCAR was not significant (p = 0.978), no imputation of missing values was conducted. Dropouts included subsequent identification of an inclusion error (e.g., no genetic test performed), women who developed OC or another cancer, or previvors who developed BC. Lost to follow-up included missing questionnaires despite multiple reminders. A total of 450 participants were enrolled in the study. There were 230 women randomized to IG and 220 to CG. 417 participants returned the baseline questionnaire t0, 398 participants completed follow-up t1, and 386 participants completed follow-up t2.

### Study population

Table 1 summarizes the baseline characteristics of the 417 women completing questionnaire t0. Women in the IG and CG were statistically comparable on the following parameters: gene variant, own cancer history, recruitment at PTGC or IBS/IBSA, time since genetic test result, own children, marital status, highest vocationally relevant degree, and employment status. Statistically significant differences occurred in age distribution and family planning, with the IG having more women aged 18–40 years and the CG having more women aged > 40 years. Fewer women in the IG had completed family planning compared to the CG.

**Table 1:** Baseline characteristics of the study population

Characteristic	TG			IG			CG			*p*-value
	n	%		n	%		n	%		
*Medical history*										
Pathogenic variant	417	100		216	100		201	100		
*BRCA1* ^*a*^	233	55.9		124	57.4		109	54.2		0.572
*BRCA2*	178	42.7		88	40.7		90	44.8		
*BRCA1* and *BRCA2*	6	1.4		4	1.9		2	1.0		
Own cancer history	417	100		216	100		201	100		
(A) No history of cancer (previvors)	292	70.0		157	72.7		135	67.2		0.219
(B) History of unilateral BC (survivors)	125	30.0		59	27.3		66	32.8		
Recruitment at	417	100		216	100		201	100		
PTGC	142	34.1		81	37.5		61	30.3		0.124
IBS/IBSA	275	65.9		135	62.5		140	69.7		
Time since genetic test result	401	100		209	100		192	100		
≤ 1 year	189	47.1		106	50.7		83	43.2		0.324
> 1 to ≤ 5 years	142	35.4		69	33.0		73	38.0		
> 5 years	70	17.5		34	16.3		36	18.8		
Own children	415	100		215	100		200	100		
Yes	243	58.6		121	56.3		122	61.0		0.329
No	172	41.4		94	43.7		78	39.0		
Family planning completed	414	100		214	100		200	100		
Yes	229	55.3		103	48.1		126	63.0		0.010*
No	159	38.4		96	44.9		63	31.5		
Not specified	26	6.3		15	7.0		11	5.5		
*Demographics*										
Mean Age (years) [SD]	39.8	[11.4]		38.2	[11.3]		41.4	[11.2]		
Age group (years)	417	100		216	100		201	100		
18–40	236	56.6		139	64.4		97	48.3		0.001*
> 40	181	43.4		77	35.6		104	51.7		
Marital status	415	100		216	100		199	100		
Married/relationship	243	58.6		123	56.9		120	60.0		0.488
Single/separated/divorced/widowed	172	41.4		93	43.1		79	39.7		
Highest vocationally relevant degree	416	100		216	100		200	100		
Academic^b^	162	38.9		82	38.0		80	40.0		0.670
Not academic^c^	254	61.1		134	62.0		120	60.3		
Employment status	415	100		216	100		199	100		
Employed^d^	284	68.4		142	65.7		142	71.4		0.219
Not employed^e^	131	31.6		74	34.3		57	28.6		

^*a*^ *one participant with a pathogenic BRCA1 variant also had a pathogenic CHEK2 variant*

^*b*^ *includes: university degree, university of applied science degree*

^*c*^ *includes: no degree, middle/intermediate school certification, final/technical high school certification*

^*d*^ *includes: full/part time employment*

^*e*^ *includes: school/training/studies, parental leave, unemployed, not able to work, retired, temporary job, not specified*

*statistically significant difference between IG and CG (Pearson’s chi-square test).

*BC: breast cancer; PTGC: posttest genetic counseling; IBS/IBSA: intensified breast surveillance (and aftercare); *

*TG: total group (IG and CG); IG: intervention group; CG: control group.*

### Decision-related outcomes

Table 2 summarizes the results for decision-related outcomes. These include decisional conflict, decision status and decision regret. Cronbach’s alpha for the DCS subscales was 0.83 for informed, 0.90 for values clarity, 0.63 for support, 0.90 for uncertainty and 0.93 for effective decision. At t0, decisional conflict was comparable for IG and CG in all mean DCS scores (total scale and subscales). At t1, scores in all DCS scales had decreased compared to t0. In the IG, all scales showed lower scores than in the CG, with statistically significant differences in the DCS total scale (primary endpoint), the informed and the support subscales. At t2, scores in all DCS scales had decreased even further. Statistically significant lower scores in the IG compared to the CG were evident for the DCS total scale, the informed, the support, and the values clarity subscales. Mean DCS total scores evaluated separately for women aged 18–40 years and > 40 years did not reveal age-related differences at any time point.

**Table 2:** Decision-related outcomes at baseline (t0), three (t1) and six months (t2) after study inclusion

		TG				IG				CG			
*t0: baseline*	Instrument / Score	n	mean (SD)	range		n	mean (SD)	range		n	mean (SD)	range	*p-value*
Decisional conflict	DCS informed	415	30.9 (21.4)	0-100		216	31.6 (22.0)	0-100		199	30.2 (20.7)	0-91.7	0.624
	DCS values clarity	412	35.6 (24.6)	0-100		213	36.1 (24.4)	0-100		199	35.0 (25.0)	0-100	0.620
	DCS support	414	27.2 (19.4)	0-83.3		215	27.0 (20.1)	0-83.3		199	27.4 (18.7)	0-83.3	0.831
	DCS uncertainty	413	49.6 (28.8)	0-100		216	50.2 (29.2)	0-100		197	48.9 (28.4)	0-100	0.630
	DCS effective decision	409	36.1 (26.4)	0-100		214	37.7 (26.9)	0-100		195	34.2 (25.8)	0-100	0.170
	DCS total	396	36.0 (20.8)	0-95.3		211	36.8 (21.3)	0-95.3		185	35.0 (20.2)	0-87.5	0.383
		n	%			n	%			n	%		
Decision status	SDM-S undecided	223	53.5			115	53.2			108	53.7		0.920
	SDM-S decided, of these	194	46.5			101	46.8			93	46.3		0.920
	IBS/IBSA only	20	4.8			14	6.5			6	3		0.095
	RRM only	11	2.6			6	2.8			5	2.5		0.853
	IBS/IBSA plus RRBSO	100	24.0			44	20.4			56	27.9		0.073
	RRM plus RRBSO	63	15.1			37	17.1			26	12.9		0.232
*t1: after 3 months*	Instrument / Score	n	mean (SD)	range		n	mean (SD)	range		n	mean (SD)	range	*p-value*
Decisional conflict	DCS informed	391	23.6 (20.3)	0-83.3		200	20.4 (19.6)	0-83.3		191	27.0 (20.5)	0-83.3	**0.001**
	DCS values clarity	392	28.0 (23.5)	0-100		201	25.8 (22.5)	0-91.7		191	30.2 (24.4)	0-100	0.082
	DCS support	391	24.3 (20.7)	0-100		202	21.5 (20.2)	0-83.3		189	27.2 (20.8)	0-100	**0.003**
	DCS uncertainty	394	40.6 (28.7)	0-100		202	39.8 (28.1)	0-100		192	41.5 (29.3)	0-100	0.617
	DCS effective decision	383	28.7 (23.8)	0-100		195	28.1 (22.8)	0-100		188	29.3 (24.9)	0-100	0.806
	DCS total	375	28.6 (20.4)	0-87.5		193	26.7 (19.3)	0-76.6		182	30.6 (21.3)	0-87.5	**0.049** ^**#**^
		n	%			n	%			n	%		
Decision status	SDM-S undecided	150	38.3			75	37.3			75	39.3		0.691
	SDM-S decided, of these	242	61.7			126	62.7			116	60.7		0.691
	IBS/IBSA only	22	5.6			14	7.0			8	4.2		0.233
	RRM only	14	3.6			6	3.0			8	4.2		0.521
	IBS/IBSA plus RRBSO	94	24.0			40	19.9			54	28.3		0.052
	RRM plus RRBSO	112	28.6			66	32.8			46	24.1		0.055
*t2: after 6 months*	Instrument / Score	n	mean (SD)	range		n	mean (SD)	range		n	mean (SD)	range	*p-value*
Decisional conflict	DCS informed	382	22.4 (19.0)	0-83.3		193	18.5 (17.3)	0–75		189	26.4 (19.8)	0-83.3	**0.000**
	DCS values clarity	380	24.8 (22.7)	0-91.7		193	21.8 (21.2)	0-91.7		187	27.9 (23.8)	0-91.7	**0.011**
	DCS support	381	22.2 (19.3)	0-83.3		192	19.1 (18.2)	0–75		189	25.4 (20.0)	0-83.3	**0.002**
	DCS uncertainty	381	36.5 (27.8)	0-100		193	34.8 (27.3)	0-100		188	38.2 (28.2)	0-100	0.249
	DCS effective decision	377	25.9 (23.2)	0-100		191	23.8 (22.3)	0-100		186	28.1 (24.0)	0-100	0.071
	DCS total	373	26.4 (19.9)	0-81.3		190	23.5 (18.7)	0-78.1		183	29.4 (20.6)	0-81.3	**0.006**
Decision regret	DRS IBS/IBSA	317	9.2 (13.1)	0–55		158	9.2 (13.1)	0–45		159	9.1 (13.0)	0–55	0.934
	DRS RRM	101	10.6 (13.7)	0–50		57	11.5 (13.2)	0–45		44	9.5 (14.4)	0–50	0.482
	DRS RRBSO	204	9.5 (12.1)	0–55		97	9.6 (12.0)	0–45		107	9.4 (12.2)	0–55	0.906
		n	%			n	%			n	%		
Decision status	SDM-S undecided	109	28.5			51	26.2			58	31.0		0.293
	SDM-S decided, of these	273	71.5			144	73.8			129	69.0		0.293
	IBS/IBSA only	23	6.0			12	6.2			11	5.9		0.911
	RRM only	13	3.4			8	4.1			5	2.7		0.441
	IBS/IBSA plus RRBSO	106	27.7			46	23.6			60	32.1		0.064
	RRM plus RRBSO	131	34.3			78	40.0			53	28.3		**0.016**

Statistical significance for any score: 2-sided Mann-Whitney-U-Test; except #: 1-sided according to study protocol [18]. Statistical significance for categorical variables: Pearson’s chi-quadrat-test. DCS: decisional conflict scale; SDM-S: stage of decision-making scale; DRS: decision regret scale; IBS/IBSA: intensified breast surveillance (and aftercare); RRM: risk-reducing mastectomy; RRBSO: risk-reducing bilateral salpingo-oophorectomy. TG: total group (IG and CG); IG: intervention group; CG: control group

At t0, 53.5% of the total group (IG and CG) classified themselves as undecided. This applied to more women aged 18–40 years than women aged > 40 years (61.4% vs. 43.1%; p = 0.000). Of those who reported choosing at least one preventive option, 26.6% had chosen one of the two risk-reducing surgeries (risk-reducing mastectomy (RRM) only or RRBSO only), 15.1% opted for both surgical options (RRM plus RRBSO), and 4.8% opted for IBS/IBSA only. At t0, IG and CG showed comparable decision status regarding all options. During follow-up (t1, t2), the proportion of undecided women continued to decrease, comparably in IG and CG, with no more significant differences between age groups. At t1, there was a non-significant trend for more women in the IG to choose RRM plus RRBSO, and more women in the CG to choose IBS/IBSA plus RRBSO. This trend strengthened at t2, when statistically significantly more women in the IG chose RRM plus RRBSO than in the CG. For all other options, IG and CG showed no statistically significant differences at t1 and t2.

The DRS for the three options displayed a Cronbach’s alpha between 0.74 and 0.83. Overall, decision regret with regard to each of the three preventive options was low, with mean scores in the lower decile of the score range for the total group, the IG, and the CG. There were no statistically significant differences between IG and CG. Women aged 18–40 years were significantly less likely than women aged > 40 years to regret the decision to have RRM (7.9 vs. 18.7; p = 0.000).

### Knowledge-related outcomes

**Fig. 2:**
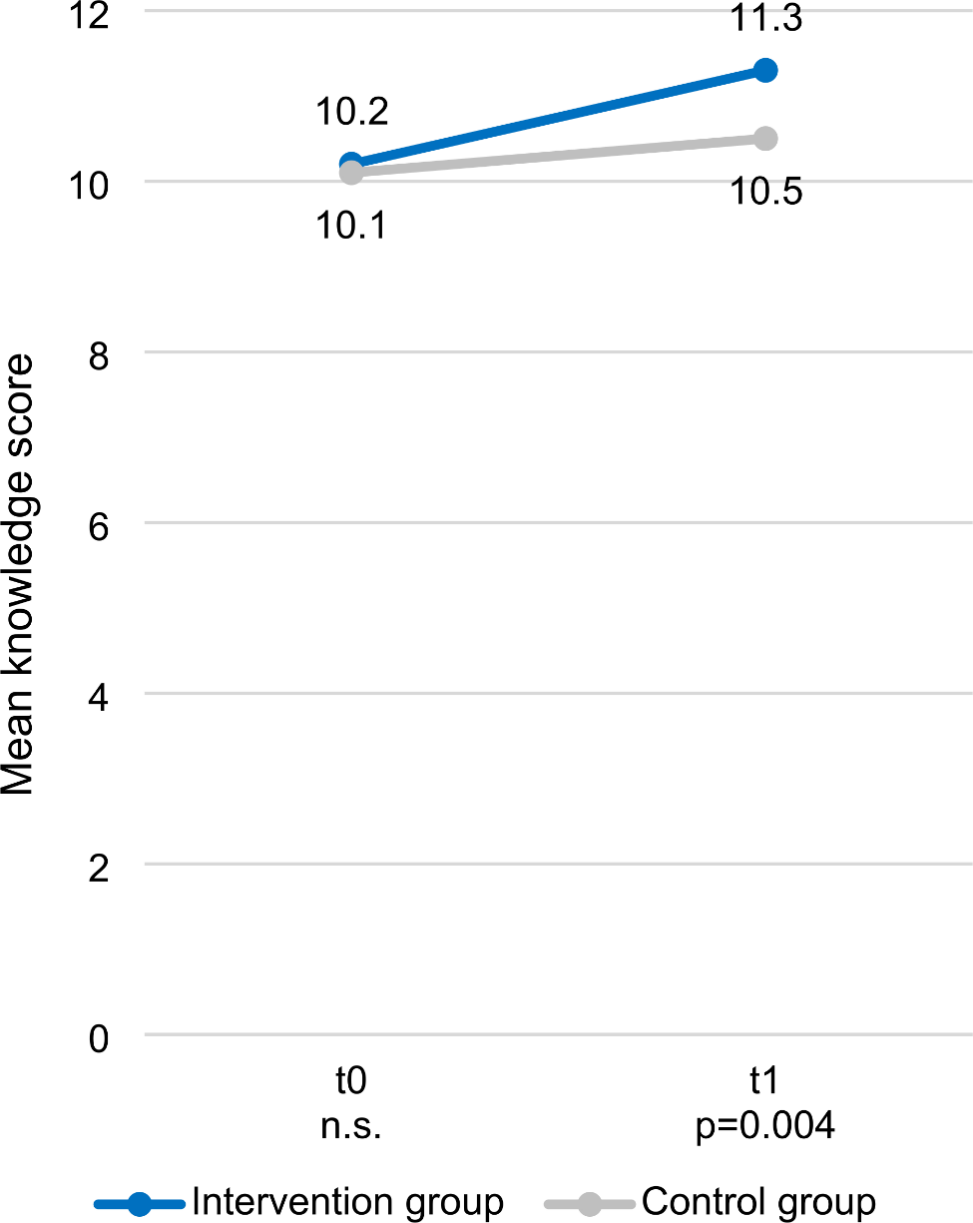
Knowledge-related outcomes at baseline (t0) and three months (t1) after study inclusion Knowledge level was determined by measuring the number of correctly rated statements per participant at baseline (t0) and three months (t1) after study inclusion. Mean scores for the IG and the CG are shown. P-values were determined by two-sided Mann-Whitney-U-test. The number of participants was as follows: at t0 the IG had n = 215, the CG n = 201; at t1 the IG had n = 205, the CG n = 191

The Cronbach’s alpha for knowledge was 0.61. The mean knowledge score of the total group was 10.2 at t0, with almost equal scores in the IG and CG (10.2 vs. 10.1; p = 0.960). At t1, the mean knowledge score in the IG had increased to 11.3. Knowledge level in the CG had increased to 10.5 at t1. As Fig. 2 demonstrates, the difference between IG and CG at t1 was statistically significant. Comparison of age groups showed significantly higher knowledge levels in women aged 18–40 years than in women aged > 40 years at both t0 (p = 0.007) and t1 (p = 0.000).

### Psychological symptoms

In the present study, Cronbach’s alpha for the HADS depression and anxiety subscales were 0.87 and 0.86, respectively. At t0, mean HADS scores in the total group were 3.7 for depressive and 7.3 for anxiety symptoms showing no statistically significant differences between IG and CG. At t1, mean HADS scores remained almost the same for depressive symptoms (3.6), but slightly decreased for anxiety symptoms (6.7) with a significantly higher mean score in the IG compared to CG (7.2 vs. 6.2, p = 0.044). At t2, both HADS scores had continued to decrease slightly, with no statistically significant differences between IG and CG. Comparison of age groups did not reveal statistically significant differences for anxiety and depressive symptoms at any survey point.

Cronbach’s alpha for the IES-R subscales was 0.86 for hyperarousal, 0.86 for intrusion and 0.89 for avoidance in the present study. At t0, self-reported distress symptoms in IG and CG were comparable in all IES-R subscales being in the lower third of the score range (hyperarousal: 8.5; intrusion: 9.8; avoidance: 11.1). At t1, mean scores in all IES-R scales had decreased slightly (hyperarousal: 7.6; intrusion: 8.3; avoidance: 10.3). IG and CG showed comparable mean scores for hyperarousal and avoidance, while the mean score for intrusion was significantly lower in CG than in IG (7.5 vs. 9.2; p = 0.033). Comparison between age groups revealed a significantly lower hyperarousal score in the 18–40 age group than in the > 40 age group at t0 (7.9 vs. 9.3; p = 0.041). This difference disappeared at follow-up t1. Post-traumatic stress-disorder (PTSD) was not present at any time.

### Acceptability and preparation for decision-making

**Fig. 3:**
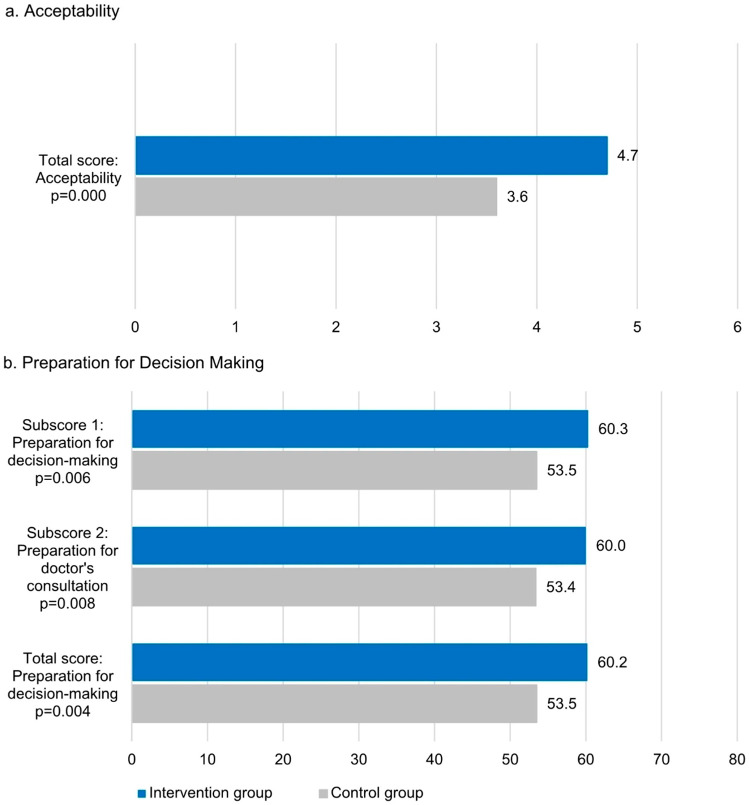
Acceptability and preparation for decision-making three months (t1) after study inclusion The perceived usefulness of the DAs (assessed by the IG) compared to written information as part of UC (assessed by the CG) was measured using (**a**) an acceptability instrument and (**b**) the preparation of decision making scale (PrepDMS) at t1 and mean scores were built. In the IG, all scales reached statistically significantly higher mean scores than in the CG. Differences were tested by Pearson’s chi-square test. The number of participants was as follows: in the acceptability survey the IG had n = 202, the CG n = 187; in the PrepDMS survey subscore 1 the IG had n = 200, the CG n = 189; in the subscore 2 survey the IG had n = 203, the CG n = 190; in the total score survey the IG had n = 200, the CG n = 189

The acceptability scale showed a Cronbach’s alpha of 0.76. Additionally, Cronbach’s alpha for the PrepDMS subscales preparation for decision-making and physician consultation were 0.93 and 0.92, respectively. Figure 3 illustrates the results for mean acceptability and PrepDMS scores at t1. IG participants showed a statistically significantly higher mean acceptability sum score and a higher rating for each individual acceptability item for the DA than CG participants did for UC. In addition, 171 of the 202 IG participants (84.7%) indicated that they would recommend the DA to other women in their situation.

All three PrepDMS scales showed statistically significantly higher mean scores in the IG than in the CG.

## Discussion

This RCT evaluated two DAs developed for women with pathogenic *BRCA1* or *BRCA2* variants in Germany with regard to their effectiveness on decision- and knowledge-related outcomes, self-reported psychological symptoms, and acceptability criteria. Compared to UC in the CG, additional DA use in the IG resulted in the following statistically significant beneficial effects: (1) decisional conflict decreased, with improvements in total scale, informed, and support subscales at t1 and t2, and values clarity subscale at t2; (2) knowledge level increased; (3) acceptability and usefulness for preparation of decision-making were rated higher by the IG for the additional DA use than by the CG for the written information usually provided as part of the UC. These results indicate that the DAs may provide valuable support to targeted women in their decision-making process.

### Study population

Study population characteristics were comparable in the IG and CG except for differences in age distribution and family planning, with the IG having more women aged 18–40 years and without completed family planning. Since most women in Germany have completed their decision to have own children at about age of 40–45 [41], the corresponding changes of both parameters seem plausible. However, given the strict adherence to double-blind randomization, the reasons for the differences in IG and CG remain unclear. Since it cannot be excluded that age and family planning status may influence the outcomes, additional analyses were performed for both age groups. Significant age-related differences were found only in a few aspects not affecting the primary outcome: women aged 18–40 years had fewer regrets regarding the decision for RRM, had higher knowledge scores before and after DA use, and showed less hyperarousal at baseline.

### Decision-related outcomes

The significant decrease in decisional conflict three months after DA use (primary outcome) compared to UC was accompanied by the feelings of being informed and supported. After six months, these effects increased even more and were accompanied by an improvement in value clarity. The results on decisional conflict are consistent with previous findings on the effectiveness of DAs specially used for women with pathogenic *BRCA1* or *BRCA2* variants [42] as well as for other health decisions [16]. The increase in the feelings of being informed and supported accompanying the decrease in decisional conflict indicates that the present DAs particularly met the needs for information about the risk situation and the trade-offs between preventive options. The additional improvement in value clarity after six months suggests that DA users became also more aware of their personal attitudes and preferences, which might also result in better preparation for informed decision-making [43]. The fact that decisional conflict, which was reduced after three months, decreased even further with DA use after six months suggests that the effect occurs early and remains sustained. This is consistent with previous findings. A decreased decisional conflict was observed as early as four or six weeks after DA use [32, 37], and persisted for 12 months [37].

The proportion of women undecided for preventive strategy strongly decreased throughout the study course, with the IG and CG having comparable decision status at baseline and after three months. This is in line with results from a study [39] that reported that women using a decision support system and those without did not differ in their decision for preventive strategy after six weeks. However, Metcalfe et al. showed that four weeks after DA use, significantly more women opted for RRM and RRBSO [32]. In our study, DA use was also associated with more women opting for RRM plus RRBSO, but this effect only reached statistical significance after six months. Considering these results together with the fact that in the CG more women tended to opting for IBS/IBSA plus RRBSO indicates that in the longer term, use of the present DAs might favor deciding for both risk-reducing surgeries.

Decision regret was low and women with and without DA did not differ in decision regret with regard to any of the preventive options. This is in line with other studies that reported on low regret or high satisfaction by women who had decided for risk-reducing surgery [44, 45] or for IBS [45]. In our study, women aged > 40 years regretted the decision for RRM to a significantly greater extent than women aged 18–40 years. Similar findings were reported within a systematic review [46], with women aged > 45 years showing greater aesthetic dissatisfaction with RRM with breast reconstruction. However, the authors also reported on data showing no age-related differences in regret, and on those in which younger women were more stressed by the reconstruction outcome, indicating uncertainty still exists.

### Knowledge-related outcomes

DA use was associated with a significant increase in knowledge about BC/OC risks and preventive options, with the IG showing a significantly higher knowledge level after three months than CG did. This is in contrast to previous RCTs on the effectiveness of DAs for women with pathogenic *BRCA1* or *BRCA2* variants showing no significant effects on knowledge-related parameters; only in one pretest-posttest trial DA use led to better risk estimates in some parts [42]. In contrast, DAs for other BC-related decisions, e.g., for early BC therapy [47] or breast reconstruction after mastectomy [48], were shown to significantly increase knowledge. One reason for the increase in knowledge with DA use in our study could be the high educational level of the study population, 38.9% of those were academics, while in Germany, only 21.4% of women aged 25 to 65 have a university degree [49]. High education level, young age and being female are associated with high health literacy [50], suggesting many participants were well qualified to read, understand, and use health information and tools like the DAs to address their own health issues. Thus, IG women had high chances to benefit from the DAs and increase their knowledge level. Another explanation for knowledge increase after DA use could be the involvement of the target group during the DA development process [17], which allowed the content to be adapted to their specific information and support needs.

### Psychological symptoms

Participants’ psychological baseline showed comparable low total scores for self-reported anxiety, depressive, and distress symptoms for IG and CG, with HADS scores in a range considered non-cases [30], and IES-R scores in the lower third of the score range. After three months, all psychological scores had decreased, but the IG had significantly higher anxiety scores than CG. This difference disappeared by six months indicating that there was no relevant long-term effects on DA users’ psychological symptoms. This is partly consistent with results from other studies examining DAs for women with pathogenic *BRCA1* and *BRCA2* variants: DA use either had no effect on anxiety [39], lowered cancer-related distress [38], or was associated with a temporarily short-term increase in distress [51] in the first month after DA use that subsequently decreased. Given the overall low level of psychological symptoms in the study population at baseline, the results on the present DAs do not appear to be clinically relevant.

### Acceptability and preparation for decision-making

The evaluated DAs were significantly better accepted and rated as more helpful for decision-making preparation by IG participants than UC by CG participants, suggesting that the DAs provide strong subjectively perceived benefit to women with pathogenic *BRCA1* and *BRCA2* variants.

### Strengths and limitations

The study has limitations that are unavoidable due to the nature of the intervention: Since DA use was voluntary and actual using patterns were not collected, it cannot be guaranteed that all IG participants used the DA. Contamination in the CG cannot be excluded, because CG participants might have gained access to the DA unintentionally. Both DA non-use in the IG and unintentional DA use in the CG could influence response behavior and contribute to potential bias. It also remains unclear to what extent participants used other sources of information and support. These could help mitigate or amplify potential effects of the DAs. Another limitation could be the unequal age distribution in the IG and CG. To check for potential bias, all main outcomes were also examined for the age groups 18–40 years and > 40 years to make possible age-related differences transparent. These revealed only minor significant differences not related to the primary endpoint. It may also be considered a limitation performing a monocentric study at a single GC-HBOC center, which could limit representativeness of the results. However, the center at the University Hospital Cologne is the largest GC-HBOC center in Germany with a broad catchment area and the largest annual number of women seeking counseling for familial BC/OC risks. Thus, it can be assumed that a wide range of women with different attitudes, needs, and experiences were able to participate in the study. A strength of this study is the RCT design including a high number of participants, which means that results of high significance for clinical care can be expected. In addition, the study was conducted in the setting of usual care (current gold standard), which corresponds to the real care situation. Comparing “usual care without DA” and “usual care plus DA” allows a clear assessment of the additional benefit that DAs can provide in the current care concept.

## Conclusions

In conclusion, the results suggest that the present DAs can support women with pathogenic *BRCA1* or *BRCA2* variants in their decision-making process by reducing decisional conflict and improving knowledge. Their high acceptability and perceived usefulness for preparation of decision-making underscores their patient-centered approach. Thus, these DAs can be a valuable addition to the current care concept for the targeted women in the German healthcare system.

## Data Availability

Only the members of the close research team have access to the trial dataset, which is not open to the public, but may be available from the corresponding author on reasonable request.

## Abbreviations

BC: Breast cancer
*BRCA1*: Breast cancer gene1
*BRCA2*: Breast cancer gene2
CG: Control group
DA(s): Decision aid(s)
DCS: Decisional conflict scale
DRS: Decision regret scale
IBS: Intensified breast surveillance
IBS/A: Intensified breast surveillance and after care
IES-R: Impact of event scale – revised
GC-HBOC: German Consortium of Hereditary Breast and Ovarian Cancer
HADS: Hospital anxiety and depression scale
IG: Intervention group
IPDAS: International Patient Decision Aid Standards
OC: Ovarian cancer
PrepDMS: Preparation for decision-making scale
PTGC: Post-test genetic counseling
RCT(s): Randomized controlled trial(s)
RRBM: Risk-reducing bilateral mastectomy
RRCM: Risk-reducing contralateral mastectomy
RRM: Risk-reducing mastectomy
RRBSO: Risk-reducing bilateral salpingo-oophorectomy
SDM-S: Stage of decision making scale
TG: Total group
UC: Usual care
